# Can *Metarhizium anisopliae* Reduce the Feeding of the Neotropical Brown Stink Bug, *Euschistus heros* (Fabricius, 1798), and Its Damage to Soybean Seeds?

**DOI:** 10.3390/jof11040247

**Published:** 2025-03-25

**Authors:** André Cirilo de Sousa Almeida, Mayara Alves Rodrigues, Heloiza Alves Boaventura, Angélica Siqueira Vieira, José Francisco Arruda e Silva, Flávio Gonçalves de Jesus, Eliane Dias Quintela

**Affiliations:** 1 Instituto Federal Goiano—Campus Urutaí, Rodovia Professor Geraldo Silva Nascimento, Km 2,5, Urutaí 75790-000, GO, Brazil; mayara-@live.com (M.A.R.); flavio.jesus@ifgoiano.edu.br (F.G.d.J.); 2 Embrapa Arroz e Feijão, Rodovia GO-462, Km 12, Fazenda Capivara, Zona Rural, CP 179, Santo Antônio de Goiás 75375-000, GO, Brazil; boaventuraheloiza@gmail.com (H.A.B.); vieira.angelica29@gmail.com (A.S.V.); jose.arruda-silva@embrapa.br (J.F.A.e.S.); eliane.quintela@embrapa.br (E.D.Q.); 3 Escola de Agronomia, Universidade Federal de Goiás, Goiânia 74690-900, GO, Brazil

**Keywords:** biological control, electropenetrography, entomopathogenic fungi, EPG

## Abstract

The fungus *Metarhizium anisopliae* is under development as a bioinsecticide for *Euschistus heros*. To further elucidate the effect of this fungus on *E. heros* behavior, we monitored the feeding activities of adults treated with the fungus at 1 × 10^8^ conidia mL^−1^ on soybean in the pod-filling stage (R5) through electropenetrography (EPG) AC-DC. We also determined the virulence of *M. anisopliae* to adults and its damage to soybean seeds. *M. anisopliae* displayed high levels of virulence to adults even at low concentrations of 5 × 10^6^ conidia mL^−1^ (98% mortality). *E. heros* females were more susceptible to *M. anisopliae* than males, exhibiting a lower LT_50_ for mycosed adults (7.1 and 9.7 days, respectively). The EPG experiment showed that fungus-treated adults spent significantly less time on probing activities (reduced by 86% at day four and ceased at day five) than untreated insects, and the number of waveform events per insect significantly decreased. This information is valuable for managing stink bugs at the field level, as it shows that even though the insect is alive, its feeding is compromised, consequently minimizing the damage inflicted to the crop. This study paves the way for further research employing entomopathogenic fungi in pest control.

## 1. Introduction

The Neotropical brown stink bug *Euschistus heros* (Fabricius, 1798) (Hemiptera: Pentatomidae) can seriously damage commodity crops, including soybean [*Glycine max* (L.) Merr.], corn (*Zea mays* L.), and cotton (*Gossypium* spp.) in Brazil [1,2]. This sucking species is also widely distributed in Argentina, Paraguay, and Uruguay [3,4].

On average, it develops from egg to adult in 25 to 32 days (5 to 7 days in the egg stage and 20 to 25 days in the nymphal stage), with the adult lifespan ranging from 75 to 116 days [5,6]. The Neotropical brown stink bug begins feeding from the second instar, and significant damage to soybean seeds occurs only after the third instar. The nymphal stages exhibit feeding behaviors similar to those of adult *E. heros*, but with some differences, particularly in the shorter duration of feeding activities compared to adults [7].

The stink bug initiates the colonization of soybean during the vegetative phase and is harmful in the reproductive phase as a pod feeder. Therefore, their feeding can directly affect yield and/or other grain-quality parameters during pod development and seed filling [1,5]. The damage results from their probing activities in soybean plants or pods when the stylet penetrates the plant tissues and injects saliva [8]. The digestive enzymes in their salivary secretions facilitate digestion, degrade tissues, and cause darkening. Feeding on pods and immature seeds can lead to malformation and abscission of pods and seeds, wrinkled or empty seeds, and darkening, with delayed maturation and reduced seed vigor [4]. In addition, the holes left by the stylets can facilitate infection by pathogenic microorganisms [9,10].

The most common method of controlling *E. heros* is application of synthetic insecticides [11]. The insecticides used to reduce stink bugs populations on soybean belong mostly to three chemical groups (neonicotinoids, organophosphates, and pyrethroids), which can be used alone or in formulated mixtures [12]. The limited number of available insecticides, combined with the frequent use of the same mode of action (often requiring 33–34 spray applications per season), has led to the selection of resistant strains [1,13,14,15,16]. Moreover, the overuse of synthetic insecticides can be detrimental to the environment, reducing natural biocontrol agents and pollinators [16,17].

Reducing synthetic chemical use in agriculture has become a global goal. Therefore, efficient and environmentally friendly multiple control strategies are needed [18,19]. Biological control [20] is a practical and ecologically friendly strategy for pest control. Entomopathogenic fungi, a proven biocontrol agent for managing stink bugs, can directly infect their host by contact through the integument, making them an important bioinsecticide for sap-sucking insects [21]. The research on stink bug management has mainly involved the fungus *Metarhizium anisopliae* (Metsch.) Sorok. (Hypocreales: Clavicipitaceae) [21,22,23].

The isolate BRM 2335 of *M. anisopliae* selected for this study has already been shown to be highly virulent against several key stink bug pests [21,23] and is under development as a mycoinsecticide for *E. heros*. Although the mode of action, virulence, and efficiency of *M. anisopliae* in managing *E. heros* have been studied, the probing behavior of this stink bug after infection with entomopathogenic fungi has yet to be elucidated.

The electropenetrography (EPG) technique is used to observe and quantify feeding behaviors otherwise hidden in the opaque food substrate [24]. EPG is an accurate and comprehensive method to evaluate the probing behavior of hemipterans [25,26] and has been employed to evaluate the probing behavior of *E. heros* [8,27]. This technique operates using an electrical circuit connecting the sucking insect to its host plant. The low electrical current flowing through the system generates waveforms that represent different probing activities (stylet penetration, salivation, and ingestion), non-probing activities, and the insect standing still or walking on the plant surface [25,26,27,28].

As fungal infection can influence host behavior [22], this technique could improve understanding of the probing behavior of piercing–sucking insects infected by an entomopathogenic fungus. The fungi take a considerable amount of time to kill their hosts—typically ranging from 2 to 10 days—depending on factors such as inoculum concentration, host species, and age. This slow process is adaptive for the pathogen, as it allows ample time to maximize nutrient extraction from the host [27]. Therefore, the probing behavior, as well as when feeding is ceased after infection, are important information for managing stink bugs, as disruptions in stylet activities can reduce the damage to productive structures of the plants. Recently, Maluta et al. [29] reported a significant disruption of the probing activities of *Dalbulus maidis* (DeLong & Wolcott) (Hemiptera: Cicadellidae) on corn after spraying *Cordyceps javanica* (Frieder. & Bally) (Hypocreales: Cordycipitaceae). The stylet activities were altered within 30 h, and these changes lasted until about 96 h.

The probing behavior of *E. heros* after entomopathogenic fungus application is not known. This pioneering study aimed to (1) determine the virulence of different *M. anisopliae* conidia concentrations to *E. heros* adults; (2) monitor the probing behavior of infected *E. heros* adults during the soybean pod-filling stage (R5) through EPG AC-DC; and (3) determine the damage to soybean pods caused by *E. heros.*

## 2. Materials and Methods

### 2.1. Stink Bug Rearing

Adult *E. heros* were collected from a soybean field at Embrapa Rice and Beans (Santo Antônio de Goiás, GO, Brazil), (16°28′00″ S, 49°17′00″ W; 823 m.a.s.l), taken to the Laboratory of Entomology, and placed in a plastic box (25 × 17 × 17 cm) lined with moistened filter paper (T 25 ± 2 °C, RH 70 ± 10%, 14-h photoperiod). Fresh green bean pods (*Phaseolus vulgaris* L.), okra fruits (*Abelmoschus esculentus* L.), mature soybean seeds (*Glycine max* L.), and raw shelled peanuts (*Arachis hypogaea* L.) were provided as food source according to Silva et al. [30].

### 2.2. Fungal Strain and Preparations

The *M. anisopliae* BRM 2335 was obtained from infected adult *Tibraca limbativentris* Stål (Hemiptera: Pentatomidae) collected from rice in a screenhouse at Embrapa Rice and Beans in 1985. The BRM 2335 isolate was identified through the sequence analysis of the elongation factor 1-alpha gene, following the protocol described in Bischoff et al. [31]. The isolate was preserved at −80 °C in the Invertebrate Fungal Collection at Embrapa Genetic Resources and Biotechnology, Brasília, DF, Brazil.

Conidia were grown on parboiled rice for 7–10 days, according to Mascarin and Quintela [32]. The conidial suspension was prepared by washing the colonized rice grains in 10 mL of sterile aqueous solution of 0.01% (*v*/*v*) Tween 80, using 50 mL plastic centrifuge tubes. The suspension was vigorously agitated on a vortex mixer for 1 min and filtered through two layers of 30 μm pore-sized nylon cheesecloth. The filtered suspension (10 mL) was vortexed again for 1 min before application, and conidial concentrations were enumerated by hemocytometer (Brightline Improved Neubauer, New Optik^®^, São Paulo, SP, Brazil) at 400× magnification. Conidial germination exceeded 98% on potato dextrose agar (PDA) after 18 h at 26 °C. Only conidia with germ tubes greater than conidial diameter were considered germinated.

### 2.3. Virulence of M. anisopliae to Adult E. heros

The fungus was tested using a control (sterile aqueous solution of 0.01% (*v*/*v*) Tween 80), as well as concentrations of 5.0 × 10^6^, 1.0 × 10^7^, 5.0 × 10^7^, and 1.0 × 10^8^ conidia mL^−1^. Carbon dioxide gas (CO_2_) was used to anesthetize adults for 15 s before fungal spraying. One milliliter of the fungal suspension was applied to 10 insects placed together in a single 60 mm Petri dish in a Potter tower calibrated at 20 psi of working pressure. After spraying, adults were kept in Gerbox-type boxes (110 × 110 × 35 mm) lined with moistened filter paper and two green bean pods. The pods were previously surface-sterilized with a sterile aqueous solution of 5% (*v*/*v*) sodium hypochlorite (2.5% NaClO) for 15 min and rinsed twice with distilled water. Assessments of live and dead insects were performed daily for 12 days. To confirm mortality resulting from fungal infection (cadavers with fungal sporulation), dead adults were transferred to Petri dishes (60 mm) with a wet cotton and maintained at room temperature. Insects were considered infected by the fungus when mycelial and conidial growth was observed on the insect cadaver.

The experiments were conducted in a completely randomized design with five replicates, each consisting of 10 insects, totaling 50 per treatment. The experiment was repeated three times. The experiments were maintained at room temperature and with a 14 h photoperiod. The temperature and relative humidity in the laboratory were monitored at 1 h intervals by two dataloggers (Hobo^®^ U12-012, Onset Computer Corp., Ltd., Bourne, MA, USA). Small variations were observed for the datalogger measurements, with an average of 25 ± 2 °C and 57 ± 15% RH.

### 2.4. Damage of E. heros Adults Treated with M. anisopliae on Soybean Seeds

Groups of five females or males of *E. heros* aged 4 to 6 days were sprayed with *M. anisopliae* at 1 × 10^8^ conidia mL^−1^ in a Potter Tower, similarly to the methodology described above. The control groups were sprayed with a sterile aqueous solution containing 0.01% (*v*/*v*) Tween 80. After spraying, each insect was transferred to a Petri dish (60 × 15 mm) with moistened filter paper and a soybean pod containing two seeds. Mortality was assessed daily for 11 days, and dead insects were transferred to Petri dishes with a wet cotton to confirm fungal infection. At 2, 4, 7, 9, and 11 days after spraying, the pod of each repetition was replaced and transferred to paper bags to dry for 7 days at room temperature. After drying, the number of feeding punctures and the damage in soybean seeds were determined by tetrazolium test, according to the methodology described in França-Neto et al. [33]. The experimental design was randomized blocks, with 15 treated males or females and untreated (control). The experiment was repeated three times.

### 2.5. Electropenetrography Studies

Soybean BRS 7470 IPRO seeds were sown weekly in soil (5 L plastic pots) and kept in a greenhouse (T 32 ± 10 °C, RH 60 ± 20%, 12 h photoperiod). After reaching the R5 stage (pod filling), stems containing pods were collected (the entire plants were too large for the Faraday cages) with a razor and placed into a small pot (0.2 L) filled with substrate [1:1–1 soil (red dystrophic oxisol), 1 sand]. The plant electrode was introduced into the substrate.

EPG data were collected using a four-channel AC/DC monitor [23] (EPG Technologies, Inc., Gainesville, FL, USA), which was connected to a computer. The recordings were made using an input impedance of 10^7^ Ohms for each of the four channels, a voltage of 50 mV alternating current (AC) via the plant electrode, and a gain set to 400× (actual gain 400 with 1× multiplier) [34]. An offset control was used to avoid rectifier fold-over and retain native waveform polarity after rectification [24]. Voltage outputs were amplified and captured at a rate of 100 Hz per channel using WinDaq DI-710 equipment (Dataq Instruments, Akron, OH, USA) and recorded on a computer with WinDaq Lite software version 3.11 (Dataq Instruments, Akron, OH, USA). The amplifiers, plants, and insects were kept inside a Faraday cage during the recordings to reduce external electrical noise.

Adults were treated with a concentration of 1 × 10^8^ conidia mL^−1^, and the control group with 0.01% *v*/*v* Tween 80, as described above. Afterward, the insects were kept in the Petri dish with two green bean pods for 48 h. Then, the insects were fasted for 5 h (without water or food) before being submitted to EPG recordings. Adhesive tape was employed to immobilize adults in a Petri dish, and dental sandpaper was used to remove the lipid layer of the insect pronotum [35]. The insects were wired by gluing one end of a gold wire (0.127 mm in diameter and 3.5 cm in length) (Sigma Aldrich, Barueri, SP, Brazil) to a copper wire (3 cm in length) soldered to a brass nail. A small loop was made at the other end of the gold wire to increase the contact area with the insect and improve electrical conductivity [36]. The gold wire was attached to the surface of the insect using a silver glue made with silver flake (Sigma Aldrich, St. Louis, MO, USA), water, and white glue (Cascorez, Jundiai, SP, Brazil) (1:1:1 wt/vol/vol) [36]. The glue dried for approximately 40 min before recording. Each stink bug was placed on a soybean pod inside the Faraday cage (as described above) and EPG-recorded for 72 h under laboratory conditions (25 ± 2 °C) and constant light [37]. Twenty adults were successfully recorded per treatment in a completely randomized design.

Waveforms were identified and named according to the nomenclature proposed by Lucini and Panizzi [6], who characterized an EPG waveform library produced by *E. heros* adult females feeding on soybean pods. The recorded waves and their biological significance are presented in Table 1. Six behaviors represented by waveforms were assessed using the variables non-probing, pathway, xylem ingestion, cell rupture, short ingestion of macerated tissues, and probable phloem sap ingestion. The five variables evaluated were waveform duration per insect (WDI), number of waveform events per insect (NWEI), waveform duration per event per insect (WDEI), percentage of recording time spent in probing (PRTP) [37,38,39,40], and final time at the last probe (FTLP).

### 2.6. Statistical Analysis

The virulence of *Metarhizium* was expressed and compared for percent of mortality, cadavers with fungal sporulation (% mycosis), and mean lethal time (LT_50_). Overall mortality and confirmed mortality curves were adjusted according to non-linear models and compared using the Chi-Square test (*p* < 0.05). To estimate the LT_50_, Gompertz and Weibull non-linear models were fitted, and values were compared by the overlap of their 95% confidence intervals (95% CI) using the Package ‘drc’ version 3.0-1 [41] in statistical software R version 4.2.2 (R Core Team 2023, available at https://www.r-project.org/ (accessed on 11 November 2024.)). Data on feeding punctures were analyzed using the Student’s *t*-test to identify statistically significant differences between groups (*p* < 0.05).

The EPG data were entered in INFEST–Insect Feeding Behavior Statistics software (available at https://arsilva.shinyapps.io/infest/ (accessed on 20 July 2024.)), which provided spreadsheets. Next, a generalized linear model (GLM) for Poisson fitted to the count data (NWEI) and GLM for Gamma were fitted for the duration variables (WDI, WDEI, PRTP, and FTLP). Differences were considered significant at α = 0.05. All analyses were performed in INFEST.

## 3. Results

### 3.1. Virulence of M. anisopliae to Adult E. heros

**Figure 1:**
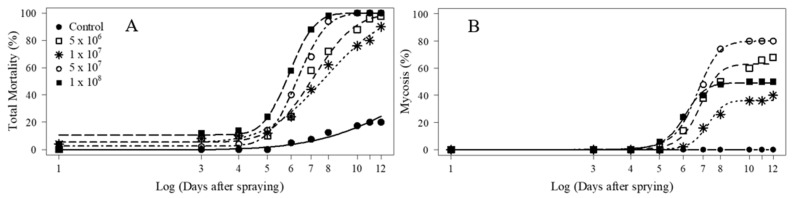
Overall (total) and confirmed mortalities (cadavers with fungal sporulation) means on different days for *Euschistus heros* adults treated with *Metarhizium anisopliae* at different concentrations. Curves were adjusted according to Logistic (**A**) and Brain–Cousens (**B**) non-linear models.

The four concentrations of *M. anisopliae* BRM 2335 caused overall and confirmed mortalities (cadavers with fungal sporulation) of adults that were statistically different from the control (Figure 1A,B; Table 2). At the lowest concentration (5 × 10^6^ conidia mL^−1^), adult mortalities were similar to those of the other concentrations (Figure 1A,B; Table 2). The fungus at 1 × 10^8^ conidia mL^−1^ killed more adults only when compared with 1 × 10^7^ conidia mL^−1^. Cadavers with fungal sporulation at 1 × 10^8^ conidia mL^−1^ were significantly higher in number than those at 1 × 10^7^ and 5 × 10^7^ conidia mL^−1^. The median lethal times (LT_50_) ranged from 5.8 to 7.6 days for all concentrations, and no differences were observed among them (Table 3).

### 3.2. Damage of E. heros Adults Treated with M. anisopliae on Soybean Seeds

**Figure 2:**
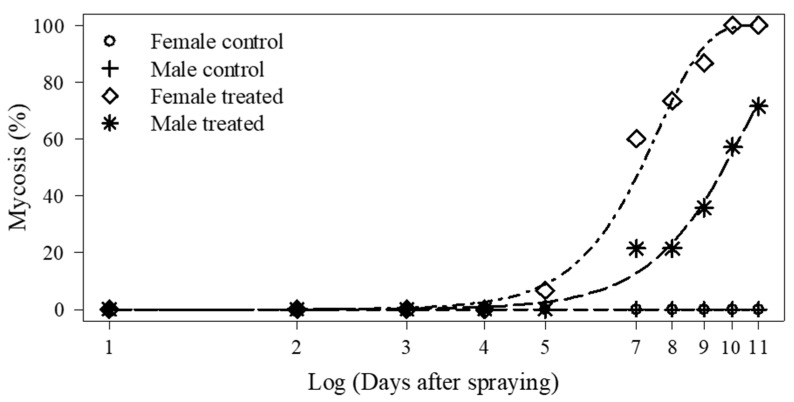
Confirmed mortality (cadavers with fungal sporulation) of female and male adult *Euschistus heros* on different days after treatment with *Metarhizium anisopliae*. Curves were adjusted according to non-linear Weibull model.

Female adult cadavers with fungal sporulation (mycosis) were significantly higher in number than those of males at 11 days after spraying (*p* < 0.001) (Figure 2). No infected adults were observed in the controls, and the counts differed between male and female groups (*p* = 0.005, *p* < 0.0001, respectively) (Figure 2). The LT_50_ was lower for females (7.1 days) than for males (9.7 days) (Table 4).

**Figure 3:**
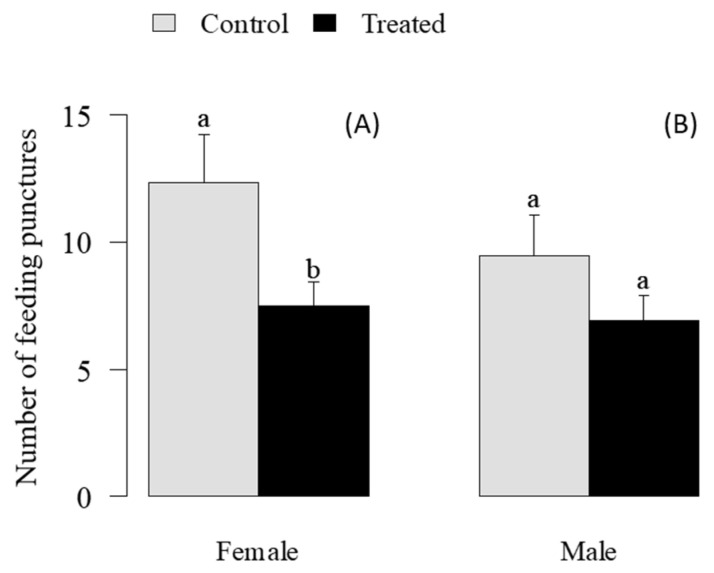
Number of feeding punctures by female (**A**) and male (**B**) adults *of Euschistus heros* in soybean seeds at 11 days after spraying of *Metarhizium anisopliae* at 1 × 10⁸ conidia mL^−1^. Different letters indicate significant differences (*p* < 0.05) by Student’s *t*-test.

The number of feeding punctures by *E. heros* females treated with *M. anisopliae* was significantly reduced compared to untreated females (t = 2.28, *p* = 0.03) at 11 days after spraying (Figure 3). However, the number of feeding punctures was similar for treated and untreated males (t = 1.25, *p* = 0.22) (Figure 3).

### 3.3. Electropenetrography

*Metarhizium anisopliae* influenced the probing behavior of *E. heros* adults on soybean pods. The NWEI was significantly reduced on treated stink bugs compared to control (Table 5). We found that *E. heros* treated with *M. anisopliae* spent significantly less time on probing activity. The WDI was shorter in xylem ingestion (waveform Eh2), laceration and maceration of endosperm (CLE–waveform Eh3a), and the short periods of ingestion of macerated tissues of endosperm (SIE–waveform Eh3b). Overall, non-probing duration (Z-Np waveform) was significantly longer in treated than in control insects.

The durations of waveform events per insect (WDEI) (Table 6) were reduced for adults infected with *M. anisopliae.* This was pronounced in non-probing (Z-Np), laceration and maceration of endosperm (CLE–waveform Eh3a), and the short periods of ingestion of macerated tissues of endosperm (SIE–waveform Eh3b). Thus, *E. heros* performed longer Z-Np, CLE, and SIE in control compared to treated insects. The PRTP was also influenced by *M. anisopliae.* The control spent more time on non-probing activities. On the other hand, the recording times in probing activities (Eh2, Eh3a, Eh3b waveforms) were lower in control compared with treated stink bugs.

**Figure 4:**
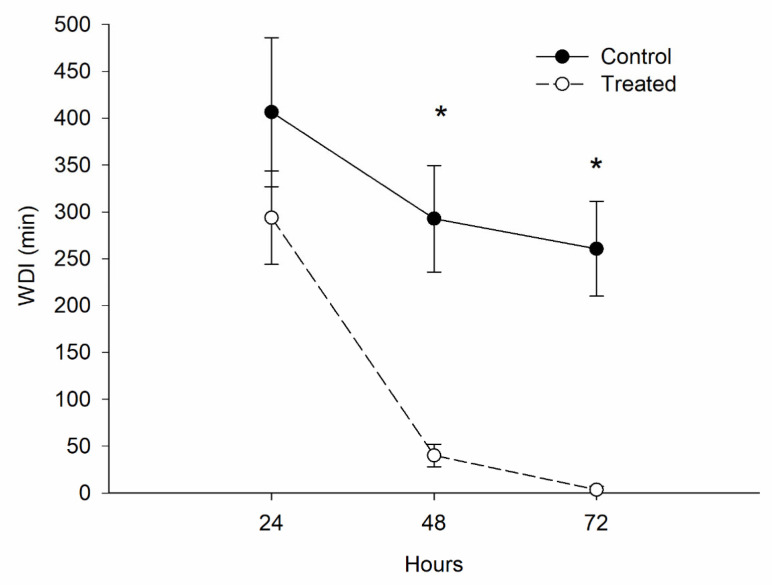
Waveform duration per insect (WDI–min) (mean ± SE) of probing activates (Eh1, Eh2, Eh3a, Eh3b, Eh4 waveforms) of *Euschistus heros* untreated (control) and treated with *Metarhizium anisopliae* feeding on soybean plants 24, 48, and 72 h after the start of recording (corresponding to 72, 96, and 120 h after fungus application, respectively). Treatments within each post-recording start time with an asterisk (*) were statistically different by F-test (*p* < 0.05) within each post-recording time.

Insects treated with *M. anisopliae* showed a reduction in WDI in probing activities (Eh1, Eh2, Eh3a, Eh3b, Eh4 waveforms) (86%) (F = 24.43, *p* < 0.001) at 48 h and (98%) (F = 10.68, *p* < 0.001) at 72 h after recording started (Figure 4).

**Figure 5:**
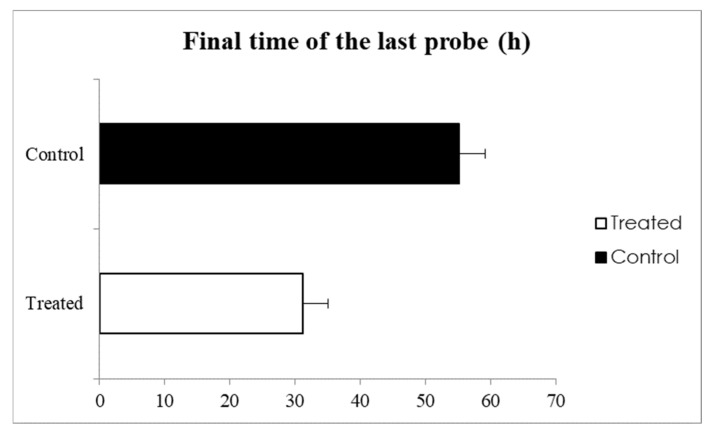
The final time of the last probe (%) of *Euschistus heros* untreated (control) and treated with *M. anisopliae* feeding on soybean plants. *p* < 0.05 is considered different by F-test.

There was a 45% reduction in the FTLP of treated stink bug adults compared to the control (Figure 5). In *E. heros* treated with *M. anisopliae*, the FTLP was 31.1 h, while in the control insects, the final time was 55.1 h (F = 18.35, *p* < 0.001).

## 4. Discussion

Among all stink bug species that damage soybean, the Neotropical brown stink bug *E. heros* is considered the most relevant pest species in all Brazil production regions [1,5], and this species is the main target of insecticide applications [42]. A soybean area of approximately 135 million ha was treated with chemical insecticides for stink bug control in the 2023/24 season [43]. Despite the large treated area, control failures or low control efficiencies have become more common [42,43,44].

The search for sustainable strategies to manage stink bug is underway with biologicals because they are host and prey of several natural enemies [1]. The most abundant egg parasitoid in soybean, *Telenomus podisi* Ashmead, 1893 (Hymenoptera, Platygastridae) is the species most commonly associated with *E. heros* in Brazil [45]. Ten commercial products containing *T. podisi* are available in Brazil for *E. heros* management [12]. Management strategies that favor the beneficial potential of parasitism must be adopted to allow more efficient natural and applied biological control. Entomopathogenic fungi, such as *M. anisopliae,* can be used to complement the egg parasitoid *T. podisi* [46].

Stink bugs, including *E. heros*, are notoriously difficult to control with entomopathogenic fungi due to the deployment of biochemical barriers, such as aldehyde production, that are quite efficient [47,48,49,50]. Despite the biochemical defenses against fungal infections, the BRM 2335 isolate of *M. anisopliae* showed high virulence against several stink bug species (Hemiptera: Pentatomidae), *E. heros*, *Oebalus poecilus* (Dallas, 1851), *Oebalus ypsilongriseus* (De Geer, 1773) (Hemiptera: Pentatomidae), *Thyanta perditor* (Fabricius, 1794) (Hemiptera: Pentatomidae), and *Tibraca limbativentris*, when compared to other *M. anisopliae* isolates, as well as *Beauveria bassiana* (Bals.) Vuill. and *Cordyceps javanica* (Hypocreales: Cordycipitaceae) [23,51]. This is supported by several studies showing that variation in host susceptibility to fungal infections depends on both species and genetic variability among isolates [52,53,54,55]

Confirming the findings of previous research, the BRM 2335 isolate of *M. anisopliae* tested in our study also displayed high levels of virulence to *E. heros* adults even at low concentrations of 5 × 10^6^ conidia mL^−1^ (98% adult mortality after 10 days). This isolate was also tested for *E. heros* under laboratory, screenhouse, and field conditions [23,56]. A limitation of *Metarhizium* for *E. heros* control is that it requires a long time to kill this pod feeder. The LT_50_ for the isolate BRM 2335 tested at 1 × 10^8^ conidia mL^−1^ (the concentration used in the EPG experiment) was 5.8 days. Despite the longer time to kill the host, the EPG experiment showed that adults treated with *M. anisopliae* spent significantly less time on probing activities than untreated insects. Furthermore, the NWEI was significantly reduced for feeding activities. Probing activities by infected adults were reduced by 86% on day 4 and ceased on day 5 (98%). EPG is an accurate method to evaluate the probing behavior of hemipterans that feed on the host’s opaque tissue [25,26]. This tool may also be useful in the applied field of stink bug management, such as to evaluate the action of insecticides affecting their feeding and survivorship [27].

Our results showed that female *E. heros* were more susceptible to *M. anisopliae* than males, exhibiting a lower LT_50_ (7.1 versus 9.7 days, respectively). This may be attributed to the fecundity of females, which drains their energy and nutrients [57]. Studies also showed that female stink bug adults accumulate more lipids than male adults, indicating higher metabolic demands and possibly longer feeding times [58,59]. This behavior explains why EPG experiments are mostly being conducted with female stink bugs. Nevertheless, additional studies are required to elucidate the physiological mechanisms underlying the susceptibility of female *E. heros* to fungal infection.

In our study, *M. anisopliae* reduced female feeding on soybean seeds, resulting in fewer punctures compared to the untreated control. These results confirmed that fungal infection can influence the damage caused by stink bugs in soybean seeds. In addition to the reduction in probing, upon penetration into the host hemocoel, *Metarhizium* fungal cells absorb nutrients and destroy host cells [60]. The fungus also secretes toxic compounds known as secondary metabolites [61]. These substances can facilitate fungal invasion [62,63] or act as immunosuppressants, compounds that resist the host’s defense [64].

Several studies have proven that *Metarhizium* can efficiently kill these insects [28]; however, little information is available about what happens to the probing behavior from the time of insect infection until death. Chen et al. [65] showed that infection by the fungus *Pandora neoaphidis* (Entomophthoromycotina: Entomophthorales) decreased stylet pathway activity and increased the time spent in non-probing activities compared with uninfected aphids. The corn leafhopper, *Dalbulus maidis,* treated with the fungus *Cordyceps javanica*, also exhibited disruption of the stylet activities in phloem and non-phloem phases 48 h after spraying [29].

Stink bugs treated with *M. anisopliae* showed a reduction (45%) in the FTLP compared with non-treated insects, i.e., treated stink bugs stopped their feeding activities earlier. This information is valuable for managing stink bugs at the field level, as it shows that even though the insect is still alive, its feeding is compromised, and consequently, the damage caused to the crop is reduced. Therefore, the slower lethal effect of the entomopathogenic fungus compared to synthetic insecticides can be minimized by changing the probing behavior of the insect treated with *M. anisopliae*. Despite the reduced time spent on probing activities by treated *Metarhizium* adults, the insect can still damage soybean seeds. One strategy to reduce the time to kill the host is mixing the entomopathogenic fungi with chemical insecticides. Sublethal or full concentrations of an insecticide can be mixed with *Metarhizium* to avoid damage to soybean by stink bugs. This strategy has proven to enhance the efficiency of entomopathogenic fungi for the control of several insect pests, in addition to reducing the selection of insects resistant to chemical insecticides [66,67,68]. Currently, this strategy (chemical + EPF) has been widely used by Brazilian producers to manage pests that are difficult to control, such as the corn leafhopper (*D. maidis*) and the whitefly *Bemisia tabaci* (Gennadius) (Hemiptera: Aleyrodidae) [69,70]. The quick action of the chemical insecticide combined with the long-lasting action of the fungi enhances the control efficiency, resulting in fewer field spraying applications [69]. Important aspects such as the compatibility between each species of fungus and the concentration of the synthetic insecticides must be taken into account.

## 5. Conclusions

In conclusion, our studies showed that Neotropical brown stink bug treated with *M. anisopliae* reduced its feeding activities by 86% at 4 days and ceased at 5 days after spraying; consequently, its damage to soybean seeds was reduced. EPG is a promising technique to evaluate the feeding behavior of stink bugs treated with entomopathogenic fungi. To further support the findings of our research, studies are underway to address the following questions: Can other fungal species and *M. anisopliae* isolates perform similarly to the highly adapted BRM 2335 isolate in reducing feeding and damage to soybean seeds by stink bugs? How do different concentrations of BRM 2335 affect the feeding activities of *E. heros*? Furthermore, this study paves the way for the further use of entomopathogenic fungi for pest control.

## Figures and Tables

**Table 1:** Waveforms recorded using electropenetrography (EPG) of *Euschistus heros* treated and untreated (control) with *Metarhizium anisopliae* fed on soybean pod and their biological significance.

Phase	Family	Type/Subtype	*Euschistus heros* Biological Meaning
Non-probing	-	Z	Insect immobile on plant surface/walking on plant surface
Pathway	P	Eh1	Penetration of stylets and salivary sheath secretion
Ingestion	I	Eh2	Xylem sap ingestion
Salivation	I	Eh3a	Cellular laceration and enzymatic maceration of seed endosperm
Ingestion	I	Eh3b	Short ingestion of macerated tissues of seed endosperm
Ingestion	I	Eh4	Short ingestion from an unknown site (probable phloem sap ingestion)

Source: Adapted from Lucini and Panizzi [7].

**Table 2:** *p*-values of the comparisons of overall (total) and confirmed mortalities (cadavers with fungal sporulation) curves for *Euschistus heros* adults after treatment with *Metarhizium anisopliae* at different concentrations. The Chi-Square test was used for *p* value calculation. Curves were considered significantly different at *p* ≤ 0.05.

Mortality				
Treatments	5.0 × 10^6^	1.0 × 10^7^	5.0 × 10^7^	1.0 × 10^8^
Control	<0.001	<0.001	<0.001	<0.001
5.0 × 10^6^	-	0.3565	0.2401	0.0663
1.0 × 10^7^	-	-	0.0460	0.0114
5.0 × 10^7^	-	-	-	0.3482
Confirmed Mortalities				
Treatments	5.0 × 10^6^	1.0 × 10^7^	5.0 × 10^7^	1.0 × 10^8^
Control	<0.001	<0.001	<0.001	<0.001
5.0 × 10^6^	-	0.0196	0.1398	0.2713
1.0 × 10^7^	-	-	0.0001	0.0754
5.0 × 10^7^	-	-	-	0.0224

**Table 3:** Estimates of parameters of non-linear models and median lethal time (LT_50_) of *Euschistus heros* adults treated with *Metarhizium anisopliae* at different concentrations (conidia mL^−1^).

Treatments (Conidia mL^−1^)	Model Parameters			LT_50_ (d) (CI95%)
	B	C	E	
5.0 × 10^6^	3.90	0.05	7.11	7.0 (5.8–8.2)
1.0 × 10^7^	2.77	0.05	7.80	7.6 (5.9–9.2)
5.0 × 10^7^	5.67	0.02	6.29	6.3 (5.4–7.1)
1.0 × 10^8^	6.42	0.10	5.89	5.8 (4.9–6.7)

Gompertz model parameters: B is the slope factor around the “E” parameter; C is the lower limit of the curve; E is the inflection point of the curve.

**Table 4:** Estimates of parameters of non-linear Weibull model and median lethal time (LT_50_) of female and male adult *Euschistus heros* treated with *Metarhizium anisopliae* at 1 × 10^8^ conidia mL^−1^.

Treatments	Model Parameters				LT_50_ (d) (CI95%)
	b	c	d	e	
Female treated	5.7	0.0	1.0	7.6	7.1 (6.5–7.7)
Male treated	5.0	0.0	1.0	10.4	9.7 (8.6–10.7)

Weibull model parameters: b is the is the slope of the curve; c is the lower limit of the curve; d is the upper limit of the curve; e is the inflection point of the curve.

**Table 5:** Number of waveform events for (NWEI) (mean ± SE) and waveform duration per insect (WDI–min) of *Euschistus heros* treated and untreated (control) with *Metarhizium anisopliae* at 1 × 10^8^ conidia mL^−1^ feeding on soybean pod-filling stage (R5) plants.

Waveform	NWEI				WDI			
	Control	Treated	F	*p* Value	Control	Treated	F	*p* Value
Z + Np-non-probing	15.9 ± 0.9	12.5 ± 0.8	6.66	0.009	3488 ± 115	3924 ± 122	6.65	0.015
Eh1-pathway	19.1 ± 0.8	12.9 ± 1.0	19.6	<0.001	77.6 ± 10.7	49.6 ± 10.3	3.51	0.065
Eh2-xylem sap ingestion	5.9 ± 0.6	1.0 ± 0.2	62.3	<0.001	189.6 ± 46.1	24.8 ± 11.7	11.93	0.016
Eh4-unknown site ingestion	2.7 ± 0.4	0.9 ± 0.4	14.8	<0.001	9.07 ± 4.9	4.23 ± 2.7	0.781	0.399
Eh3a-CLE	26.5 ± 1.2	14.9 ± 0.9	52.3	<0.001	550.2 ± 106.4	239.4 ± 50.4	8.20	0.007
Eh3b-SIE	13.6 ± 0.9	4.6 ± 0.5	74.2	<0.001	5.42 ± 1.60	1.26 ± 0.4	8.08	0.007

CLE–cellular laceration of seed endosperm, SIE–short ingestion of macerated tissues of seed endosperm. *p* < 0.05 is considered different by F-test.

**Table 6:** Waveform duration per event per insect (WDEI-min) (mean ± SE) and percentage of recording time spent in probing (PRTP-%) of *Euschistus heros* untreated (control) and treated with *Metarhizium anisopliae* at 1 × 10^8^ conidia mL^−1^ feeding on soybean pod-filling stage (R5) plants.

Waveform		WDEI			PRTP			
	Control	Treated	F	*p* Value	Control	Treated	F	*p* Value
Z + Np-non-probing	356.1 ± 81.2	1115.2 ± 401.8	6.80	0.014	80.8 ± 2.8	90.3 ± 2.6	6.55	0.015
Eh1-pathway	4.44 ± 0.4	3.23 ± 0.4	3.73	0.628	1.8 ± 0.2	1.2 ± 0.2	3.22	0.082
Eh2-xylem sap ingestion	34.5 ± 10.2	15.0 ± 7.8	1.29	0.263	4.4 ± 1.0	0.6 ± 0.2	14.7	0.007
Eh4-Phloem ingestion	2.0 ± 0.4	1.0 ± 0.6	1.29	0.264	0.3 ± 0.1	0.1 ± 0.0	0.825	0.370
Eh3a-CLE	21.4 ± 2.6	13.7 ± 2.2	4.55	0.041	12.8 ± 2.4	5.6 ± 1.1	8.23	0.004
Eh3b-SIE	0.5 ± 0.1	0.2 ± 0.0	4.68	0.038	0.2 ± 0.0	0.1 ± 0.0	8.77	0.005

CLE—cellular laceration of seed endosperm, SIE—short ingestion of macerated tissues of seed endosperm. *p* < 0.05 is considered different by F-test.

## Data Availability

The original contributions presented in the study are included in the article, further inquiries can be directed to the corresponding author.
